# Evaluation of reference genes for transcript analyses in *Komagataella phaffii* (*Pichia pastoris*)

**DOI:** 10.1186/s40694-023-00154-1

**Published:** 2023-03-29

**Authors:** Mihail Besleaga, Gabriel A. Vignolle, Julian Kopp, Oliver Spadiut, Robert L. Mach, Astrid R. Mach-Aigner, Christian Zimmermann

**Affiliations:** 1 Institute of Chemical, Environmental and Bioscience Engineering, TU Wien, Gumpendorfer Strasse 1a, 1060 Wien, Austria; 2 Center for Health and Bioresources, Competence Unit Molecular Diagnostics, AIT Austrian Institute of Technology GmbH, 1210 Vienna, Austria

**Keywords:** *Komagataella phaffii*, *Pichia pastoris*, RT-qPCR, Relative transcript analysis, Transcriptomics, Housekeeping genes

## Abstract

**Background:**

The yeast *Komagataella phaffii* (*Pichia pastoris*) is routinely used for heterologous protein expression and is suggested as a model organism for yeast. Despite its importance and application potential, no reference gene for transcript analysis via RT-qPCR assays has been evaluated to date. In this study, we searched publicly available RNASeq data for stably expressed genes to find potential reference genes for relative transcript analysis by RT-qPCR in *K. phaffii*. To evaluate the applicability of these genes, we used a diverse set of samples from three different strains and a broad range of cultivation conditions. The transcript levels of 9 genes were measured and compared using commonly applied bioinformatic tools.

**Results:**

We could demonstrate that the often-used reference gene ACT1 is not very stably expressed and could identify two genes with outstandingly low transcript level fluctuations. Consequently, we suggest the two genes, RSC1, and TAF10 to be simultaneously used as reference genes in transcript analyses by RT-qPCR in *K. phaffii* in future RT-qPCR assays.

**Conclusion:**

The usage of ACT1 as a reference gene in RT-qPCR analysis might lead to distorted results due to the instability of its transcript levels. In this study, we evaluated the transcript levels of several genes and found RSC1 and TAF10 to be extremely stable. Using these genes holds the promise for reliable RT-qPCR results.

**Supplementary Information:**

The online version contains supplementary material available at 10.1186/s40694-023-00154-1.

## Background

The ascomycetous yeast *Komagataella phaffii* (previously *Pichia pastoris* [1]) is routinely used for heterologous protein expression due to its fast growth rates and cheap cultivation conditions—compared to mammalian cell cultures, and its capability to secrete high levels of recombinant proteins [2]. Moreover, a large genetic toolbox is available for *K. phaffi* making it an interesting workhorse for the production of recombinant proteins in academia as well as the biopharmaceutical industry [3]. *K. phaffi* was also suggested to be used as a model organism for yeasts as it has undergone a slower evolution than *Saccharomyces cerevisiae* and retained more characteristics of ancient yeasts and other metazoan organisms [4]. Consequently, different aspects of general biology and especially the protein production and secretion pathway have been studied over the years (reviewed in [3–8]). In many of these studies, reverse transcription quantitative PCR (RT-qPCR) analyses are used to determine the transcript levels of native and heterologous genes [9–20].

RT-qPCR is a routinely used method for the quantification of individual transcripts in biological samples. In brief, first, the RNA is extracted and then reverse transcribed yielding cDNA. This cDNA is then used as template in a qPCR reaction. The design and choice of primers determine which sequence is to be amplified and therefore quantified. Generally, qPCR assays can be used to calculate the absolute number of targets in a sample, when a set of serial standard dilutions is used to infer a standard curve. However, RT-qPCR assays are mostly performed to compare the abundance of certain transcripts among two or more samples. By an appropriate choice of samples, we can evaluate the influences of different stimuli, cultivation time, developmental state, and many other factors on the transcript abundance of the chosen genes of interest.

Currently, different mathematical methods are used to calculate the relative transcript levels and compare different samples. The commonly used “Delta–delta method” was first described in the Applied Biosystems User Bulletin No. 2 (P/N 4303859). It presumes an identical and perfect efficiency of target and reference genes. In contrast, the model of Pfaffl takes different efficiencies into account [21]. The ‘efficiency calibrated mathematical method for the relative expression ratio in real-time PCR’ was partially published in an internal magazine of Roche (Biochemica No. 2 2001). According to Pfaffl [21], the Roche model is mathematically hard to follow but delivers identical results as Pfaffl’s method. The standard curve based method of Larionov et al. does not need to take efficiencies into account, as it is based on dilution standards [22]. Notably, all these calculation methods rely on the usage of a reference value to compare different samples. Current state-of-the-art is the usage of internal reference values i.e., reference genes. These are genes with constant transcript levels in all samples. Therefore, their abundance in a sample is a representation of the sample itself. The more reference genes transcript is present, the more RNA was isolated and successfully transcribed. Normalization to reference genes aims to mitigate or even nullify differences in the relative abundance of the different types of RNA (rRNA, tRNA, mRNA, etc.) or in the efficiency of the reverse transcription among the different samples.

Based on this central role for the calculation of relative transcript levels, the choice of reference genes is of utmost importance and must be carefully validated. This is stressed in the widely accepted ‘Minimum Information for Publication of Quantitative Real-Time PCR Experiments’ (MIQE) guidelines [23] and was previously discussed and demonstrated in other fungi such as *S. cerevisiae* [24], *Aspergillus* spp. [25, 26], and *Trichoderma reesei* [27]. The MIQE guidelines further state that at least two reference genes should be used for accurate normalization [23]. In *K. phaffi*, ACT1 (encoding for the cytoskeleton-monomer, actin) is often used as reference gene in RT-qPCR assays [9, 10, 12, 14–20]. To our knowledge, this gene is used in the *K. phaffi* community out of habit and due to the lack of validated reference genes.

In this study, we assessed several publicly available RNA-Seq data sets and ranked all annotated genes according to their variation among the samples. We manually curated this list and chose eight stably expressed genes. To test and validate these genes and ACT1, we compiled a test set of 35 independent samples. These samples cover a broad range of typical and atypical cultivation conditions and stresses and originate from three different *K. phaffi* strains. The transcript levels of the eight potential reference genes and ACT1 were measured in all samples and evaluated with routinely used tools (i.e., the comparative Delta Ct method [28], BestKeeper [29], Normfinder [30], Genorm [31], and RefFinder [32]).

## Results

### Identification of stably expressed genes by the assessment of RNA-Seq data

To identify potential reference genes in *K. phaffii*, we searched for stably expressed genes in publicly available RNASeq data (Table 1). The used data sets cover a variety of different culturing conditions and sampling timepoints of the strain GS115. We compared the data sets “Glycerol”, “Methanol”, “Glycerol and Methanol” and “YNBE” to “Glucose” each and rigorously filtered (robust base mean coverage and low log2 Fold Change, details in the Materials and Methods section) for genes with stable expression in each of those four comparisons (Additional file 1: Table S1). Next, we narrowed the gene list down to those genes that appeared in each comparison, giving us a final set of 38 genes (Additional file 2: Table S2).

**Table 1:** RNA-Seq datasets used in this study

Sample ID	Accession	Platform	Run details	Culturing condition, sampling timepoint	Sample set	References
GSM4225310: B_10	SRX7415644	Illumina NovaSeq 6000	13.8G bases, 4.1 Gb	Glucose / Bench-scale batch growth fermentation at 10 h	Glucose	[33]
GSM4225313: M_12	SRX7415647	Illumina NovaSeq 6000	10.6G bases, 3.2 Gb	Methanol induction / Methanol growth 12 h	Methanol	[33]
GSM4225314: M_24	SRX7415648	Illumina NovaSeq 6000	11.5G bases, 3.5 Gb	Methanol induction / Methanol growth 24 h	Methanol	[33]
GSM4225311: G_3	SRX7415645	Illumina NovaSeq 6000	11.4G bases, 3.3 Gb	Glycerol growth at 3 h	Glycerol	[33]
GSM4025512: GS115.0G_A	SRX6691833	NextSeq 500	236.7 M bases, 96.1 Mb	24 h glycerol	Glycerol	[34]
GSM4025513: GS115.0G_B	SRX6691834	NextSeq 500	233.7 M bases, 95 Mb	24 h glycerol	Glycerol	[34]
GSM4025515: GS115.24G_B	SRX6691836	NextSeq 500	270.2 M bases, 109.4 Mb	48 h glycerol	Glycerol	[34]
GSM4225312: GM_2	SRX7415646	Illumina NovaSeq 6000	14.2G bases, 4.2 Gb	Glycerol and methanol mixture feed culture at 2 h	Glycerol and Methanol	[33]
GSM4025517: GS115.24M_A	SRX6691838	NextSeq 500	206.1 M bases, 83.4 Mb	24 h glycerol + 24 h methanol	Glycerol and methanol	[34]
GSM4025518: GS115.24M_B	SRX6691839	NextSeq 500	284.4 M bases, 114.6 Mb	24 h glycerol + 24 h methanol	Glycerol and methanol	[34]
GSM5155619: GS115	SRX10307452	HiSeq X Ten	4.7G bases, 1.8 Gb	YNBE (Yeast Nitrogen Base Ethanol) media for 14 h	YNBE	[35]

### Comparison and evaluation of refences genes

Next, we manually picked 8 genes to be experimentally tested for stable expression in *K. phaffii* (Table 2). We decided to exclude genes without homologs in *S. cerevisiae* and without gene description. Further, we omitted all genes whose products are potentially somehow involved in or influenced by mechanisms of protein expression or secretion (e.g., ER-residing proteins). Hence, we focused on genes whose products are involved in nuclear processes (e.g., ribosome synthesis, epigenetics, transcription). We also included the currently often used reference gene, ACT1 in our evaluation experiment, although ACT1 was not identified as stably expressed (Additional file 2: Table S2).

**Table 2:** Potential reference genes to be empirically evaluated

Nucleotide ID	Homolog in *S. cerevisiae*	Description
XM_002490190.1	RPD3	Histone deacetylase
XM_002490251.1	TAF10	Subunit (145 kDa) of TFIID and SAGA complexes
XM_002490943.1	ARX1	Shuttling pre-60S factor
XM_002491604.1	ARP9	Component of both the SWI/SNF and RSC chromatin remodeling complexes
XM_002491925.1	VMA6	Subunit d of the five-subunit V0 integral membrane domain of vacuolar H±ATPase
XM_002492119.1	RSC1	Component of the RSC chromatin remodeling complex
XM_002493755.1	TFC7	One of six subunits of the RNA polymerase III transcription initiation factor complex (TFIIIC)
XM_002493961.1	RPP1	Subunit of both RNase MRP and nuclear RNase P
XM_002492401.1	ACT1	Component of the cytoskeleton

To test the applicability of the potential reference genes we compiled a sample set from three *K. phaffii* strains covering a broad range of cultivation conditions, such as different carbon sources, cultivation methods, cultivation stage and different stress types (Table 3). The samples were designed to represent typical cultivation conditions in *K. phaffii* experiments. Thus, we reason that genes found to be expressed stably in these samples might be suitable reference genes for future transcript analyses. Notably, no biological replicates of the cultivation conditions were performed, as we did not aim to determine the concrete transcript levels and performance of the genes in each sample. In contrast, our goal was to create a sample set with a large diversity to generate data on the overall applicability and robustness of the genes.

**Table 3:** Samples used for reference gene evaluation

Sample	Strain	Medium	Cultivation time and/or Condition
1	CBS 7435	YNBM	0 h
2	CBS 7435	YNBM, 1% Glu	8 h
3	CBS 7435	YNBM, 1% Glu	8 h
4	CBS 7435	YNBM, 1% Gly	8 h
5	CBS 7435	YNBM, 1% Gly	8 h
6	CBS 7435	YNBM, 1% MeOH	8 h
7	CBS 7435	YNBM, no C-source	8 h
8	CBS 7435	LB	8 h
9	CBS 7435	DMSZ, 1% Glu	8 h
10	CBS 7435	BMGY, 1% Glu	8 h
11	CBS 7435	YNBM, 1% Glu	pH = 3.0, 8 h
12	CBS 7435	YNBM, 1% Glu	pH = 7, 8 h
13	CBS 7435	YNBM, 1% Glu	37 °C, 8 h
14	CBS 7435	YNBM, 1% Glu	18 °C, 8 h
15	CBS 7435	YNBM, 1% Glu	2% EtOH, 8 h
16	CBS 7435	YNBM, 1% Glu	0.5 M NaCl, 8 h
17	CBS 7435	YNBM, 1% Glu	2% H_2_O_2_, 8 h
18	CBS 7435	YNBM, 1% Glu	10 µg/mL Zeocin, 8 h
19	CBS 7435	YNBM, 1% Glu	No agitation, 8 h
20	GS115	YNBM, 1% Glu	8 h
21	GS115	YNBM, 1% Gly	8 h
22	GS115	YNBM, 1% MeOH	8 h
23	GS115	LB	8 h
24	GS115	DMSZ, 1% Glu	8 h
25	GS115	BMGY, 1% Glu	8 h
26	BSYBG11	BSM, 1% MeOH	1 h
27	BSYBG11	BSM, 2% Gly	1 h
28	BSYBG11	BSM, 1% MeOH	8 h
29	BSYBG11	BSM, 2% Gly	8 h
30	BSYBG11	BSM, starvation	8 h after Gly depletion
31	BSYBG11	BSM	End Batch
32	BSYBG11	BSM	End Batch + MeOH pulse
33	BSYBG11	BSM	Fed-Batch, Methanol feed
34	BSYBG11	BSM	Start Fed Batch (Gly)
35	BSYBG11	BSM	End Fed Batch (Gly)

**Fig. 1:**
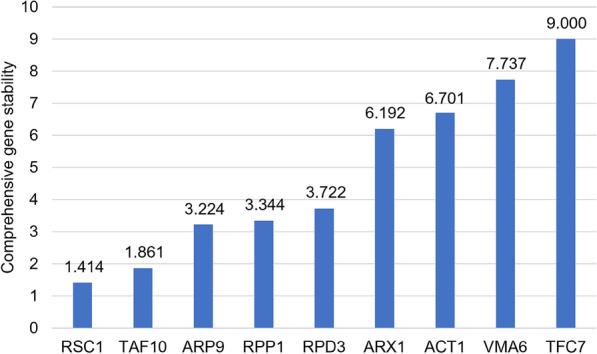
Comprehensive gene stability of the tested genes. This value is an dimension-less integration of the stability values calculated by the comparative Delta Ct method [28], BestKeeper [29], Normfinder [30], Genorm [31], and RefFinder [32] and ranks the tested genes accordingly (see also Table A3, Additional file 3: Table S3). Lower values represent higher stability in the tested samples (Table 3)

The samples were subjected to RT-qPCR analyses measuring the transcript levels of the potential reference genes. The obtained Ct values (Additional file 3: Table S3) were entered into the RefFinder [32] online tool at http://blooge.cn/RefFinder/. This tool integrates the four commonly used tools, the comparative Delta Ct method [28], BestKeeper [29], Normfinder [30] and Genorm [31] and calculates and comprehensive value based on the results of the four individual results (Additional file 3: Table S3 and Fig. 1). Out of the nine tested genes, RSC1 and TAF10 had the lowest variability and thus the most stable transcript levels. In contrast, ACT1 had a substantially higher “comprehensive gene stability” value (Fig. 1 and Additional file 3: Table S3) and was thus less stably expressed in the tested samples.

## Discussion

The choice of a reference gene is an essential and crucial part of a transcript analysis via RT-qPCR. The abundance of a reference gene’s transcript shall represent the overall amount of isolated RNA and reverse transcribed cDNA. Consequently, using genes with fluctuating transcript levels is detrimental and prevents a reliable transcript analysis. In this study, we filtered publicly available RNA Seq data set from *K. phaffii* GS115 for genes with a low overall log2 Fold Change in different data set comparison and manually revised the gene list to obtain potential reference genes. We then tested and evaluated the applicability of these genes experimentally by creating a diverse set of samples from three *K. phaffii* strains under a broad range of cultivation conditions. The transcript levels of the genes were then compared and evaluated using five broadly used tools for reference gene evaluation.

The obtained results demonstrate that the often-used ACT1 gene is not as stably expressed as other genes (Fig. 1) in our tested samples (Table 3). The stability value (calculated with geNORM finder) of ACT1 of 0.656 lies above the suggested cut-off of 0.5 according to [36]. Further, the average standard deviation (calculated with BestKeeper) of ACT1 lies with 1.029 just above the suggested cut-off of 1.0 according to [29]. This is in accordance with previous findings in *S. cerevisiae* [24], where ACT1 was also shown to be unsuitable as reference gene. Consequently, we strongly discourage the usage of this gene as reference gene in future RT-qPCR assays in *K. phaffii*.

The transcript levels of the genes RSC1 and TAF10 exhibited generally low log2 Fold Changes during the comparison of the RNASeq data (Additional file 1: Table S1) and had the lowest “comprehensive gene stability” in our experimental gene evaluation assay. RSC1 is part of the RSC chromatin remodeling complex, while TAF10 is a subunit of both the TFIID complex and the SAGA complex. Both complexes are involved in the gene transcription by the RNA Polymerase II [37]. Based on their biological roles, it comes to no surprise that RSC1 and TAF10 are expressed stably. We consider both genes as suitable reference genes and recommend the simultaneous usage (according to the MIQE guidelines [23]) for relative transcript analyses in *K. phaffii*.

## Materials and methods

### Assessment of RNA-Seq data

To search for genes with stable transcript levels, we used 11 publicly available RNA-Seq data sets from *K. phaffii* GS115. These datasets were derived from three studies and encompass 9 different sampling conditions (Table 1). The RNA-Seq datasets were downloaded from the Sequence Read Archive (SRA) [38] of the National Center for Biotechnology Information (NCBI), with a total of 67.429 G bases and 20.597 Gb. We grouped the different data sets into five sample sets based on the added carbon source (“Glucose”, “Glycerol”, “Glycerol and Methanol”, “Methanol”, “YNBE”) according to the description in the original studies (Table 1). The raw reads were inspected using FastQC v0.11.5 and then analyzed and quality trimmed using Trimmomatic [39]. We extracted a reference transcriptome using gffread v0.12.7 [40] from the reference genome of* K. phaffii* GS115 [41, 42]. Next, we used salmon 1.4.0 [43] to create a salmon index on the reference transcriptome and quantified each of the datasets, including the –gcBias flag to account for the effects of sample specific biases such as fragment-level GC bias. The quantification results were imported into the R environment and analyzed with the DESeq2 package [44], the tximport package [45], and the Bioconductor package [46].

Next, we compared the following sample sets using DEseq2: glycerol vs. glucose, glycerol and methanol vs. glucose, methanol vs. glucose and YNBE vs. glucose. For each comparison we calculated the Log fold change shrinkage (LFC) the expressed genes. These were then filtered for genes with a base mean coverage of > 500 and < 3000, to avoid false positives and potential sequencing artifacts, and a log2 Fold Change of < 0.05 and > -0.05 to screen for genes with low changes of the transcript levels within the different data sets (same carbon source, different time points). The obtained gene lists were then compared and filtered for genes appearing in each comparison to obtain a list of genes with stable transcript levels in different carbon sources compared to “Glucose”.

### Fungal strains and cultivation conditions

The *K. phaffi* strains CBS 7435, GS115 and BSYBG11 (constitutively expressing a recombinant protein) were pre-cultivated in Yeast nitrogen base media (YNBM) for 20 h at 30 °C, 230 rpm in a shaking incubator (Multitron, Infors HT, Basel, Switzerland). The YNBM consisted of potassium phosphate buffer (pH 6.0), 0.1 M; Yeast Nitrogen Base w/o Amino acids and Ammonia Sulfate (BD Difco, Difco Laboratories Incorporated, part of BD, Franklin Lakes, NJ, USA), 13.4 g/L; (NH_4_)_2_SO_4_, 10 g/L; biotin, 400 mg/L; glucose, 20 g/L [47]. For GS115, Histidine was added to cultivation resulting in a final concentration of 20 mg/L. Once the complete substrate in the initial culture was consumed (at line-determined via Cedex measurements, as described previously [48]), the cultures were used as inoculum (representing 10% of the final volume) for further cultivations. The pre-cultures were used to inoculate shake flasks containing specific medium and conditions (Table 3, samples 2–29). Samples were taken after 1 h or 8 h. Sample 1 was taken from the pre-culture of CBS 7435 directly after glycerol depletion, whereas sample 30 was taken 8 h after glycerol depletion from the pre-culture of BSYBG11.

The low salt lysogenic broth (LB) medium consisted of tryptone, 10.0 g/L; NaCl, 5.0 g/L; yeast extract, 5.0 g/L. The BMGY media consisted of yeast extract, 10.0 g/L; peptone, 20.0 g/L; potassium phosphate buffer (pH 6.0), 0.1 M; Yeast Nitrogen Base w/o Amino acids and Ammonia Sulfate (Difco), 13.4 g/L; biotin, 400 mg/L; glycerol, 10.0 g/L. The DSMZ medium consisted of yeast extract, 3.0 g/L; malt extract, 3.0 g/L; peptone from soybeans, 5.0 g/L; glucose, 10.0 g/L. Basal salt medium (BSM) consists of 85% (v/v) phosphoric acid, 26.7 mL/L; CaSO_4_*2H_2_O, 1.17 g/L; K_2_SO_4_, 18.2 g/L; MgSO_4_*7H_2_O, 14.9 g/L; KOH, 4.13 g/L; glycerol, 20 g/L supplied with trace elements [49].

The strain BSYBG11 constitutively expressing a recombinant protein was also cultivated in a Minifors 2 bioreactor system (max. working volume: 2 L; Infors HT, Bottmingen, Switzerland). The cultivation offgas flow was analyzed online using offgas sensors—IR for CO_2_ and ZrO_2_ based for O_2_ (Blue Sens Gas analytics, Herten, Germany). Process control and feeding was performed using EVE software (Infors HT, Bottmingen, Switzerland). The pH was monitored using a pH-sensor EasyFerm Plus (Hamilton, Reno, NV, USA). During the cultivations pH was kept constant at 5.0 and was controlled with base addition only (12.5% NH_4_OH), while acid (10% H_3_PO_4_) was added manually, if necessary. Temperature was kept constant at 30 °C. Aeration was carried out using a mixture of pressurized air and pure oxygen at 2 vvm to keep dissolved oxygen (dO_2_) always higher than 30%. The dissolved oxygen was monitored using fluorescence dissolved oxygen electrode Visiferm DO (Hamilton, Reno, NV, USA). At the end of the batch phase, represented by a sudden drop in CO_2_ signal and a parallel increase in the dO_2_, methanol pulse (0.5% v/v, supplied with basal media trace element stock solution) was added to the bioreactors for metabolism adaptation (Table 3, samples 32–33). After 24 h from adaptation pulse, fed-batch cultivation started and lasted 42 h. Mixed-feed (80 g/L methanol mixed with 400 g/L glycerol) was used for samples 32 and 33 (Table 3) while for samples 34 and 35, (Table 3) a derepressed feeding strategy was applied by setting feeding rate at a limiting level.

### RNA extraction

Approx. 0.1 g of yeast cells were resuspended in 1 ml RNAzol RT (Sigma-Aldrich) and lyzed using a Fast-Prep-24 (MP Biomedicals, Santa Ana, CA, USA) with 0.5 g of glass beads (1 mm diameter) twice at 6 m/s for 30 s. Samples were incubated at room temperature for 5 min and then centrifuged at 12,000 g for 5 min. 650 µl of the supernatant were mixed with 650 µl ethanol and RNA isolated using the Direct-zol RNA Miniprep Kit (Zymo Research, Irvine, CA, USA) according to the manufacturer’s instructions. This Kit includes a DNAse treatment step. The concentration and purity were measured using a NanoDrop ONE (Thermo Fisher Scientific, Waltham, MA, USA).

### RT-qPCR assays

500 ng of isolated total RNA was reverse transcribed using the LunaScript RT SuperMix (NEB) according to the manufacturer’s instructions. The resulting cDNA was diluted 1:50 and 2 µl were used as template in a 15 µl reaction using the Luna Universal qPCR Master Mix (NEB) according to the manufacturer’s instructions. Used primers are listed in (Additional file 4: Table S4). All reactions were performed in technical duplicates on a Rotor-Gene Q system (Qiagen, Hilden, Germany).

## Supplementary Information

**Additional file 1: Table S1.** Genes with low log2FoldChanges in the different data set comparisons.

**Additional file 2: Table S2.** Genes with consistent low log2FoldChanges in all data set comparisons.

**Additional file 3: Table S3.** Ct_values_evaluation.

**Additional file 4: Table S4.** Primers used in this study.

## Data Availability

All data generated or analyzed during this study are included in this published article and its supplementary information files.

## Abbreviations

BMGY: Buffered glycerol-complex medium
BSM: Basal salt medium
cDNA: Copy DNA
DMSZ: Deutsche Sammlung von Mikroorganismen und Zellkulturen
ER: Endoplasmic reticulum
LB: Lysogenic broth, or Luria broth
MIQE: Minimum information for publication of quantitative real-time PCR experiments
mRNA: Messenger RNA
NCBI: National Center for Biotechnology Information
RNase MRP: RNAse for mitochondrial RNA processing
RNASeq: RNA sequencing
rNRA: Ribosomal RNA
RSC: Remodeling the structure of chromatin
RT-qPCR: Reverse transcription quantitative PCR
SAGA: Spt-Ada-Gcn5 acetyltransferase
SRA: Sequence read archive
SWI/SNF: SWItch/sucrose non-fermentable
TFIID: Transcription factor II D
TFIIIC: Transcription factor III C
tRNA: Transfer RNA
YNBE: Yeast nitrogen base ethanol
YNBM: Yeast nitrogen base media

## References

[1] Kurtzman CP (2009). Biotechnological strains of *Komagataella* (*Pichia*) *pastoris* are *Komagataella*
*phaffii* as determined from multigene sequence analysis. J Ind Microbiol Biotechnol.

[2] Freyre FM, Vázquez JE, Ayala M, Canaán-Haden L, Bell H, Rodríguez I (2000). Very high expression of an anti-carcinoembryonic antigen single chain Fv antibody fragment in the yeast *Pichia pastoris*. J Biotechnol.

[3] Fischer JE, Glieder A (2019). Current advances in engineering tools for *Pichia pastoris*. Curr Opin Biotechnol.

[4] Bernauer L, Radkohl A, Lehmayer LGK, Emmerstorfer-Augustin A (2021). *Komagataella*
*phaffii* as emerging model organism in fundamental research. Front Microbiol..

[5] Bustos C, Quezada J, Veas R, Altamirano C, Braun-Galleani S, Fickers P (2022). Advances in cell engineering of the *Komagataella*
*phaffii* platform for recombinant protein production. Metabolites.

[6] Peña DA, Gasser B, Zanghellini J, Steiger MG, Mattanovich D (2018). Metabolic engineering of *Pichia pastoris*. Metab Eng.

[7] Raschmanová H, Weninger A, Knejzlík Z, Melzoch K, Kovar K (2021). Engineering of the unfolded protein response pathway in *Pichia pastoris*: enhancing production of secreted recombinant proteins. Appl Microbiol Biotechnol.

[8] Liu C, Gong J-S, Su C, Li H, Li H, Rao Z-M (2022). Pathway engineering facilitates efficient protein expression in *Pichia pastoris*. Appl Microbiol Biotechnol.

[9] Guerfal M, Ryckaert S, Jacobs PP, Ameloot P, Van Craenenbroeck K, Derycke R (2010). The HAC1 gene from *Pichia pastoris*: characterization and effect of its overexpression on the production of secreted, surface displayed and membrane proteins. Microb Cell Fact.

[10] Sjöblom M, Lindberg L, Holgersson J, Rova U (2012). Secretion and expression dynamics of a GFP-tagged mucin-type fusion protein in high cell density *Pichia pastoris* bioreactor cultivations. Adv Biosci Biotechnol.

[11] Sha C, Yu X-W, Li F, Xu Y (2013). Impact of gene dosage on the production of lipase from *Rhizopus chinensis* CCTCC M201021 in *Pichia pastoris*. Appl Biochem Biotechnol.

[12] Zhu T, Hang H, Chu J, Zhuang Y, Zhang S, Guo M (2013). Transcriptional investigation of the effect of mixed feeding to identify the main cellular stresses on recombinant *Pichia pastoris*. J Ind Microbiol Biotechnol.

[13] He J, Ma X, Zhang F, Li L, Deng J, Xue W (2015). New strategy for expression of recombinant hydroxylated human collagen α1 (III) chains in *Pichia pastoris* GS 115. Biotechnol Appl Biochem.

[14] Yang H, Zhai C, Yu X, Li Z, Tang W, Liu Y (2016). High-level expression of Proteinase K from *Tritirachium** album* Limber in *Pichia pastoris* using multi-copy expression strains. Protein Expr Purif.

[15] Aw R, McKay PF, Shattock RJ, Polizzi KM (2017). Expressing anti-HIV VRC01 antibody using the murine IgG1 secretion signal in *Pichia pastoris*. AMB Express.

[16] Tredwell GD, Aw R, Edwards-Jones B, Leak DJ, Bundy JG (2017). Rapid screening of cellular stress responses in recombinant *Pichia pastoris* strains using metabolite profiling. J Ind Microbiol Biotechnol.

[17] Ito Y, Terai G, Ishigami M, Hashiba N, Nakamura Y, Bamba T (2020). Exchange of endogenous and heterogeneous yeast terminators in *Pichia pastoris* to tune mRNA stability and gene expression. Nucleic Acids Res.

[18] Hou C, Yang Y, Xing Y, Zhan C, Liu G, Liu X (2020). Targeted editing of transcriptional activator MXR1 on the *Pichia pastoris* genome using CRISPR/Cas9 technology. Yeast.

[19] Lin N-X, He R-Z, Xu Y, Yu X-W (2021). Oxidative stress tolerance contributes to heterologous protein production in *Pichia pastoris*. Biotechnol Biofuels.

[20] Dou W, Zhu Q, Zhang M, Jia Z, Guan W (2021). Screening and evaluation of the strong endogenous promoters in *Pichia pastoris*. Microb Cell Fact.

[21] Pfaffl MW (2001). A new mathematical model for relative quantification in real-time RT-PCR. Nucleic Acids Res.

[22] Larionov A, Krause A, Miller W (2005). A standard curve based method for relative real time PCR data processing. BMC Bioinformatics.

[23] Bustin SA, Benes V, Garson JA, Hellemans J, Huggett J, Kubista M (2009). The MIQE guidelines: minimum information for publication of quantitative real-time PCR experiments. Clin Chem.

[24] Teste M-A, Duquenne M, François JM, Parrou J-L (2009). Validation of reference genes for quantitative expression analysis by real-time RT-PCR in *Saccharomyces cerevisiae*. BMC Mol Biol.

[25] Archer M, Xu J (2021). Current practices for reference gene selection in RT-qPCR of *Aspergillus*: outlook and recommendations for the future. Genes.

[26] Suleman E, Somai BM (2012). Validation of *hisH4* and *cox5* reference genes for RT-qPCR analysis of gene expression in *Aspergillus flavus* under aflatoxin conducive and non-conducive conditions. Microbiol Res.

[27] Steiger MG, Mach RL, Mach-Aigner AR (2010). An accurate normalization strategy for RT-qPCR in *Hypocrea*
*jecorina* (*Trichoderma*
*reesei*). J Biotechnol.

[28] Silver N, Best S, Jiang J, Thein SL (2006). Selection of housekeeping genes for gene expression studies in human reticulocytes using real-time PCR. BMC Mol Biol.

[29] Pfaffl MW, Tichopad A, Prgomet C, Neuvians TP (2004). Determination of stable housekeeping genes, differentially regulated target genes and sample integrity: BestKeeper–Excel-based tool using pair-wise correlations. Biotechnol Lett.

[30] Andersen CL, Jensen JL, Ørntoft TF (2004). Normalization of real-time quantitative reverse transcription-PCR data: a model-based variance estimation approach to identify genes suited for normalization, applied to bladder and colon cancer data sets. Can Res.

[31] Vandesompele J, De Preter K, Pattyn F, Poppe B, Van Roy N, De Paepe A (2002). Accurate normalization of real-time quantitative RT-PCR data by geometric averaging of multiple internal control genes. Genome Biol.

[32] Xie F, Xiao P, Chen D, Xu L, Zhang B (2012). miRDeepFinder: a miRNA analysis tool for deep sequencing of plant small RNAs. Plant Mol Biol.

[33] Zhang C, Ma Y, Miao H, Tang X, Xu B, Wu Q (2020). Transcriptomic analysis of *Pichia*
*pastoris* (*Komagataella*
*phaffii*) GS115 during heterologous protein production using a high-cell-density fed-batch cultivation strategy. Front Microbiol.

[34] Brady JR, Whittaker CA, Tan MC, Kristensen DL, Ma D, Dalvie NC (2020). Comparative genome-scale analysis of *Pichia pastoris* variants informs selection of an optimal base strain. Biotechnol Bioeng.

[35] Gupta A, Krishna Rao K, Sahu U, Rangarajan PN (2021). Characterization of the transactivation and nuclear localization functions of *Pichia pastoris* zinc finger transcription factor Mxr1p. J Biol Chem.

[36] Hellemans J, Mortier G, De Paepe A, Speleman F, Vandesompele J (2007). qBase relative quantification framework and software for management and automated analysis of real-time quantitative PCR data. Genome Biol.

[37] Lee TI, Causton HC, Holstege FC, Shen WC, Hannett N, Jennings EG (2000). Redundant roles for the TFIID and SAGA complexes in global transcription. Nature.

[38] Leinonen R, Sugawara H, Shumway M (2011). The sequence read archive. Nucleic Acids Res.

[39] Bolger AM, Lohse M, Usadel B (2014). Trimmomatic: a flexible trimmer for Illumina sequence data. Bioinformatics.

[40] Pertea G, Pertea M (2020). GFF utilities: GffRead and GffCompare. F1000Res.

[41] Sturmberger L, Chappell T, Geier M, Krainer F, Day KJ, Vide U (2016). Refined *Pichia pastoris* reference genome sequence. J Biotechnol.

[42] Valli M, Tatto NE, Peymann A, Gruber C, Landes N, Ekker H (2016). Curation of the genome annotation of *Pichia*
*pastoris* (*Komagataella*
*phaffii*) CBS7435 from gene level to protein function. FEMS Yeast Res.

[43] Patro R, Duggal G, Love MI, Irizarry RA, Kingsford C (2017). Salmon provides fast and bias-aware quantification of transcript expression. Nat Methods.

[44] Love MI, Huber W, Anders S (2014). Moderated estimation of fold change and dispersion for RNA-seq data with DESeq2. Genome Biol.

[45] Soneson C, Love MI, Robinson MD (2015). Differential analyses for RNA-seq: transcript-level estimates improve gene-level inferences. F1000Res.

[46] Zhu A, Ibrahim JG, Love MI (2019). Heavy-tailed prior distributions for sequence count data: removing the noise and preserving large differences. Bioinformatics.

[47] Dietzsch C, Spadiut O, Herwig C (2011). A dynamic method based on the specific substrate uptake rate to set up a feeding strategy for *Pichia pastoris*. Microb Cell Fact.

[48] Gundinger T, Kittler S, Kubicek S, Kopp J, Spadiut O (2022). Recombinant protein production in *E. coli* using the phoA expression system. Fermentation.

[49] Spadiut O, Dietzsch C, Herwig C (2014). Determination of a dynamic feeding strategy for recombinant *Pichia pastoris* strains. Methods Mol Biol (Clifton, NJ).

