# Supplementary material for: Evaluation of reference genes for transcript analyses in Komagataella phaffii (Pichia pastoris)
Source: Fungal Biol Biotechnol. 2023 Mar 29;10:7. doi: 10.1186/s40694-023-00154-1 (PMC10061771; doi:10.1186/s40694-023-00154-1)
Supplement: Supplementary file 4 — Additional file 4: Table S4. Primers used in this study. [file 40694_2023_154_MOESM4_ESM.pdf]

Evaluation of reference genes for transcript analyses in *Komagataella phaffii* (*Pichia pastoris*)

Mihail Besleaga, Gabriel A. Vignolle, Julian Kopp, Oliver Spadiut, Robert L. Mach, Astrid R. Mach-Aigner, Christian Zimmermann

Additional File 4 – Primers used in this study

Name	Sequence (5’ – 3’)
ACT1_fwd	CCTTGAGGCTTCTGGTATCGA
ACT1_rev	CTGGTGGAGCAGAAATCTTGAC
TAF10_fwd	CCTCACATTTCTATGCCTTCCC
TAF10_rev	TCACTGATAAACTTTTGGGTAGC
RPD3_fwd	ACGTGGACGTTTGAGTCAGG
RPD3_rev	CTGGAGGTACATAGTTAAGCTGAACG
ARX1_fwd	GTCCATCACTTCCAAGCCTTC
ARX1_rev	CTGTTGGTGATCTCGGACATTC
ARP9_fwd	CAAGGCTGCGATGAGTTAGTAAG
ARP9_rev	CTGCATCAGCCTTAGATCTCTTG
VMA6_fwd	CCCTAAGTGTAGCTACCGATATTG
VMA6_rev	CTCAGGAGTTAACTCAGTACCAATTG
RSC1_fwd	CACGATGCCTGCTGATGTGG
RSC1_rev	GGGTACATACACTGGAGGACCTC
TFC7_fwd	CGGATGATGAGTATGAAGACGTTTAC
TFC7_rev	ACGTTGGCTCAATATTGGACATC
RPP1_fwd	TGATCCATCCCAGTGTCAAGG
RPP1_rev	CGTTGAAATGCTACCAGCTGG
